# Mitogenomic Insights on the Phylogeny and Evolution of Lynx Spiders (Araneae, Oxyopidae)

**DOI:** 10.1002/ece3.72192

**Published:** 2025-09-18

**Authors:** Dan Fu, Lijuan Liu, Changjun Wu, Yufa Luo

**Affiliations:** ^1^ Key Laboratory of Wetland Biodiversity of the Jianhu Basin of Shaoxing, School of Life and Environmental Sciences Shaoxing University Shaoxing China

**Keywords:** Cenozoic, maternal egg‐guarding behaviors, mitogenome, phylogenetic relationships, phylogenomics

## Abstract

Lynx spiders (Oxyopidae Thorell, 1869) have high diversity and widespread distribution (nine genera and 447 species currently described worldwide). They are important predators of many arthropod pests in agriculture and forestry. Although the taxonomy of lynx spiders has received attention, there is a lack of studies on their phylogeny, as well as the evolution of important ecological traits. Herein, we inferred the phylogenetic relationships and evolutionary history of lynx spiders using mitochondrial genomes, analyzed the characteristics of their mitogenomes, and tested the evolutionary pattern of maternal egg‐guarding (EG) behaviors of lynx spiders. Our results suggest that the genera *Oxyopes* Latreille, 1804, *Hamataliwa* Keyserling, 1887, *Hamadruas* Deeleman‐Reinhold, 2009, and *Peucetia* Thorell, 1869 are all the monophyletic groups, but *Tapponia* Simon, 1885 is not. Their phylogenetic relationships are shown as (*Peucetia*, (*Oxyopes*, (*Hamadruas*, (*Tapponia*, *Hamataliwa*)))). The family Oxyopidae is probably originated around 73.5 million years ago (Ma; 67.1–80.4 Ma) during the Late Cretaceous. We found only one gene arrangement pattern in the mitogenomes of Oxyopidae. Within the 13 protein‐coding genes, only the COX1 gene change is positively affected by natural selection. The ancestral state reconstructions inferred the evolutionary process of three EG behaviors of lynx spiders. This study has advanced our understanding of the phylogenetic relationships among lynx spiders and their mitogenomic evolution, as well as the likely evolutionary pattern of oxyopid maternal EG behaviors.

## Graphical abstract

This paper studied the phylogenetic relationships among lynx spiders and their mitogenomic evolution, as well as the likely evolutionary pattern of oxyopid maternal egg‐guarding (EG) behaviors.

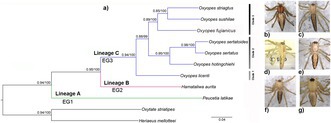

## 1 Introduction

The family Oxyopidae Thorell, 1879, commonly known as lynx spiders, is easily recognized by its unique hexagonal eye arrangements, tapered abdomens, and legs with conspicuous and erect black spines (Brady 1964; Dondale and Redner 1990; Brady and Santos 2005; Jocqué and Dippenaar‐Schoeman 2006). Lynx spiders are a diverse and widespread spider group currently including nine genera and 447 described species worldwide (10 species of *Hamadruas* Deeleman‐Reinhold, 2009 in Asia; 90, 284, and 47 species of *Hamataliwa* Keyserling, 1887, *Oxyopes* Latreille, 1804, and *Peucetia* Thorell, 1869 globally, respectively; one of *Hostus* Simon, 1898 in Madagascar; one of *Pseudohostus* Rainbow, 1915 in southern Australia; seven of *Schaenicoscelis* Simon, 1898, and three of *Tapinillus* Simon, 1898 in South America; and four of *Tapponia* Simon, 1885 in Southeast Asia) (World Spider Catalog 2025). However, the phylogeny and evolution of lynx spiders remain poorly understood (Griswold 2010), which hinders their taxonomic and systematic studies and applications as an effective biological control agents in agroecosystems.

The eggs of lynx spiders are laid in tough silken sacs, which are anchored to twigs and leaves, and the mother remains to guard them until the eggs hatch (Forster and Forster 1973; Cutler et al. 1977). Lynx spiders exhibit a variety of maternal egg‐guarding (EG) behaviors: (1) species of *Peucetia* suspend their egg sacs with tangled silks extending to nearby leaves or stems and hold the sacs under them (EG1; Whitcomb 1962; Fink 1987; our observations); (2) *Hamataliwa*, *Hamadruas*, and *Tapponia* yield their egg sacs attached to dried leaves hanged on by lines (EG2; van Niekerk and Dippenaar‐Schoeman 1994; Lo et al. 2024; our observations); and (3) the species of *Oxyopes* directly attaches their egg sacs to the plants (EG3; Lo et al. 2024; our observations). However, the evolutionary process of the three maternal EG behaviors has not yet been tested. Fossil records suggest the family Oxyopidae is a relatively young lineage that appeared likely during the Paleogene (about 43–47.8 million years ago, Ma; Wunderlich 2004). Therefore, lynx spiders are likely a good animal group for studies on the recent evolution of maternal care behaviors.

Studies on systematics need a robust phylogeny, and also the phylogenies are only one of the steps necessary for testing hypotheses related to historic ecological and behavioral evolution patterns. Mitogenomes are a good molecular marker for the studies of animal phylogeny due to their gene‐content conservation, and rapid evolutionary rate (Boore 1999). However, to date, the mitogenomes of only three species of lynx spiders were determined (KM272950 by Pan et al. 2016, MW832846 by Li et al. 2022, and unpublished MT741489 from GenBank). Here, we sequenced the mitogenomes of six oxyopid species and applied mitogenomic data to explore the phylogeny, the timing and the mitogenomic evolution, as well as the maternal EG behavioral evolution of lynx spiders. We produced the robust phylogenies of the sampled oxyopid taxa using the mitogenomes and the COX1 gene for assessing their lineage relationships and testing the evolutionary pattern of the maternal EG behaviors of lynx spiders.

## 2 Materials and Methods

### 2.1 Sample Collection and DNA Extraction

Six species of three oxyopid genera were collected from China between 2016 and 2023 (Table 1; Table S1). The specimens were identified by morphology and stored in 95% ethanol at −20°C in the Zoological Museum of Shaoxing University, Shaoxing, China, for sequencing and phylogenetic analyses. Total genomic DNA was extracted from the cephalothorax and legs of the sample using a TIANamp Genomic DNA kit (DP304‐02) and sent to Personalbio Biotechnology Company (Shanghai, China) for high‐throughput sequencing. Additional sequences of the mitogenomes from *Peucetia latikae* Tikader, 1970 (Li et al. 2022), *Oxyopes licenti* Schenkel, 1953 (Li et al. 2022), and *Oxyopes sertatus* L. Koch, 1878 (Pan et al. 2016) were downloaded from GenBank (Table 1; Table S1).

**TABLE 1:** Oxyopid samples from China: Species name, specimen voucher, sample collection locality with coordinates, maternal egg‐guarding behavior, GenBank accession number, mitogenomic size, reference of assembly and reference literature.

Species	Voucher	Locality	Maternal egg‐guarding behavior/reference	Accession number/references	Size (bp)	Reference of assembly (taxon/accession number)
*Peucetia latikae*	—	China, Hunan	EG1/our observations	MW832846/Li et al. (2022)	14,566	—
*Hamataliwa aurita*	LCGW1	China, Jiangxi (N: 27°45′12′′, E: 117°40′36′′)	EG2/our observations	PV743049/this study	14,890	*O. hupingensis* /MK518391
*Oxyopes licenti*	—	China, Gansu	EG3/our observations	MT741489/Li et al. (2022)	14,431	—
*Oxyopes hotingchiehi*	LCGW5	China, Jiangxi (N: 25°46′21′′, E: 114°58′10′′)	EG3/our observations	PV692091/this study	14,428	*O. sertatus* /KM272950
*Oxyopes sertatus*	2013 phcnjoxse	China, Nanjing	EG3/our observations	KM272950/Pan et al. (2016)	14,442	—
*Oxyopes sertatoides*	LCGW6	China, Jiangxi (N: 25°45′33′′, E: 114°57′24′′)	EG3/our observations	PV692092/this study	14,447	*O. sertatus* /KM272950
*Oxyopes fujianicus*	LCGW7	China, Jiangxi (N: 25°45′27′′, E: 114°58′11′′)	EG3/our observations	PV692090/this study	14,405	*O. sertatus* /KM272950
*Oxyopes sushilae*	LCGW4	China, Jiangxi (N: 25°46′21′′, E: 114°58′10′′)	EG3/our observations	PV692094/this study	14,412	*O. sertatus* /KM272950
*Oxyopes striagatus*	LCGW2	China, Jiangxi (N: 25°45′27′′, E: 114°58′12′′)	EG3/our observations	PV692093/this study	14,477	*O. sertatus* /KM272950

*Note:* EG1–EG3 represent the three types of maternal egg‐guarding behaviors of the sampled oxyopids in this study.

### 2.2 Sequencing, Assembly and Annotation

The Illumina PE150 libraries were constructed and sequenced on an Illumina Novoseq 6000 platform (Illumina, USA) using the total genomic DNA. The low‐quality reads were removed from the raw sequencing data by fastp v0.23.4 using the default parameters (Chen et al. 2018). The remaining clean reads were used to assemble into the mitogenome using the Geneious Prime v2024.0.5 (Kearse et al. 2012) with reference to the previously published mitogenome of the closely related species (Table 1). Gene annotations of the assembled mitogenomes were performed on the MITOS website (https://usegalaxy.org, Bernt et al. 2013). Sequences of the annotated 13 protein‐coding genes (PCGs) were reviewed by searching the NCBI non‐redundant protein sequence database using BLAST (Altschul et al. 1997). We determined the 22 tRNA gene sequences using the MITOS website and ARWEN v1.2 (Laslett and Canbäck 2008).

### 2.3 Analyses of Gene Arrangement and PCGs Evolutionary Rate

Gene arrangement analyses of the mitogenomes for the retrolateral tibial apophysis (RTA) spider clade were performed using CREx (http://mitos.bioinf.uni‐leipzig.de/index.py, Bernt et al. 2007). The CREx was used for pairwise comparisons of gene order in different mitogenomes to identify the mitochondrial gene rearrangements (MGTs) and to infer the evolutionary process, with the MGTs including transposition (T), reverse transposition (rT), inversion (I), and tandem duplication‐random loss (TDRL). For each PCG of all oxyopid species, the Ka (non‐synonymous substitutions) and Ks (synonymous substitutions) values and the ratios (Ka/Ks) were calculated between the outgroup *Oxytate striatipes* L. Koch, 1878 and all included oxyopid species using DnaSP v5.10.01 (Librado and Rozas 2009). A Ka/Ks value of more than one indicates that the effect of natural selection on gene changes is positive, whereas a Ka/Ks value of less than one indicates that natural selection negatively influences gene sequence changes (Kryazhimskiy and Plotkin 2008).

### 2.4 Molecular Phylogenetic Analyses

Phylogenies of the oxyopid taxa were reconstructed based on the mitochondrial genome/gene sequences from this study, Pan et al. (2016), Wang et al. (2018), Lo et al. (2024), and GenBank (Table S1). Alignments were performed on the online version of MAFFT v.7.0 (Katoh and Standley 2013) using the algorithms G–INS–i for highly conserved sequences (13 PCGs) and Q–INS–i for sequences with more variable regions (12S and 16S); all other settings were left as default. All aligned fragments were visually inspected, and PCGs were translated into amino acids to reduce possible mismatches. Alignments of the 12S and 16S gene sequences were refined using Gblocks v0.91b (Castresana 2000). For each gene, possible saturation of substitution types was checked by plotting the number of transitions (Ti) and transversions (Tv) versus TN93 distance using DAMBE (Xia and Xie 2001). We first inferred the phylogeny of nine species within the three genera (*Oxyopes*, *Hamataliwa*, and *Peucetia*) of lynx spiders using the concatenated 13 PCGs and two rRNA sequences (Table S1). Second, we constructed the phylogeny of 17 oxyopid species (seven *Oxyopes*, four *Hamataliwa*, three *Tapponia*, one *Hamadruas*, and two *Peucetia*) using the mitochondrial COX1 gene sequences (Table S1). Phylogenetic analyses were conducted with the Bayesian inference (BI) and maximum‐likelihood (ML) approaches. *Oxytate striatipes* and *Heriaeus mellotteei* Simon, 1886 from Thomisidae Sundevall, 1833, closely related to the Oxyopidae, were used as outgroups (Table S1).

The BI analyses were performed in MrBayes v.3.2 (Ronquist et al. 2012). We first conducted the BI analysis using the concatenated 13 PCGs and two rRNAs dataset. The best‐fitting substitution model for each gene partition (Table S2) was determined by jModelTest (Posada 2008). The Markov chain was run for 7000 generations. Further, we conducted the BI analysis with the more oxyopid taxa using the COX1 dataset; we ran the chain for 507,000 generations. The chains of both datasets were sampled every 100 generations. Tracer v1.5 (Rambaut and Drummond 2009) was employed to monitor the mixing of the chains. In each BI analysis, a 50% majority rule consensus tree was computed after discarding the first 20% of trees as burn‐in.

The ML analyses were implemented in the online phylogenetic tool W‐IQ‐TREE (Trifinopoulos et al. 2016). We used the Bayesian information criterion and the FreeRate heterogeneity to select the best‐fitting substitution model for each gene partition (Kalyaanamoorthy et al. 2017). TIM3+F+I for 12S and ND4L; GTR+F+G4 for 16S and COX3; TIM+F+G4 for ATP6, COX2, Cytb, ND2 and ND4; HKY+F+G4 for ATP8 and ND3; TIM+F+I+G4 for COX1 and ND1; GTR+F+I+G4 for ND5; and TVM+F+G4 for ND6 were used in the ML analysis based on the concatenated 13 PCGs and two rRNAs dataset; and GTR+F+I+G4 was used in the ML analysis based on the COX1 dataset. The number of iterations since the last best tree found (*c*) and the perturbation strength (*p*) were set to 1000 and 0.3, respectively. We used the ultrafast bootstrap (UFBoot, Minh et al. 2013) with 1000 of the maximum number of iterations and 0.99 of the minimum correlation coefficient to estimate the support of phylogenetic tree branches.

### 2.5 Divergence Time Estimation

The concatenated 13 PCGs and two rRNAs dataset was used to date the trees of oxyopid species. For each gene, we first checked possible saturation of substitution types by plotting the number of transitions and transversions versus TN93 distance using DAMBE. Divergence time was estimated in BEAST v1.8.2 (Drummond and Rambaut 2007) using an uncorrelated lognormal relaxed molecular clock model. Analyses with a Yule and birth–death prior were repeated to assess the sensitivity of the results to tree prior specification. The birth–death model outperformed the Yule model according to the marginal likelihood estimated by Stepping‐stone sampling. Hence, for subsequent analyses, we focused on the chronogram resulting from the birth–death prior. Partitioned strategies (Brandley et al. 2005) were used in BEAST analyses. The best‐fitting substitution model for each partition/gene was selected based on the Bayesian information criterion using jModelTest (GTR+I+G for 12S, 16S, COX1, COX2, COX3, Cytb, ND1, ND2, ND4, ND5 and ND6; GTR+G for ND3; HKY+I+G for ND4L and ATP6; and HKY+G for ATP8). The MCMC chains were run for 30 million generations with a sampling frequency of 1000. The first 20% of the yielded trees were discarded as burn‐in, and the remaining trees were used to compute the maximum clade credibility tree in TreeAnnotator v1.8.0. Outputs were reviewed in Tracer v1.7 to check all effective sample sizes (> 200).

For the molecular clock analyses, we used secondary calibrations, taken from the divergent time (80–90 Ma) of Oxyopidae and Thomisidae from the fossil‐based chronograms of spiders of Magalhaes et al. (2020), Shao et al. (2023), and Wolff et al. (2022), as corresponding to our tree (see Table S3). The 13 species of Pisauridae Simon, 1890, Lycosidae, Thomisidae and Salticidae Blackwall, 1841 were used as outgroups. Their gene sequences were available from our study and GenBank (Table S1).

### 2.6 Maternal Egg‐Guarding Behavior Reconstructions

The EG1 behavior of *P. latikae* , the EG2 behavior of *H. aurita* Zhang, Zhu & Song, 2005, and the EG3 behaviors of *O. hotingchiehi* Schenkel, 1963, *O. sertatoides* Xie & Kim, 1996, *O. fujianicus* Song & Zhu, 1993, *O. sushilae* Tikader, 1965, *O. striagatus* Song, 1991, *O. licenti* , and *O. sertatus* were observed in this study (see Table 1). The oxyopid sister groups Thomisidae and Ctenidae carry their egg sacs in the chelicerae and build the nursery webs in which to hang the sacs just prior to the emergence of spiderlings (Fisher et al. 1999; Thorp et al. 2014; our observations). The sister group Lycosidae actively transports the egg sac on the mother's spinnerets and the young spiderlings on her abdomen (Murphy et al. 2006; our observations). Comparative analyses found that the oxyopid EG1–EG3 behaviors were unrelated to those of Thomisidae, Ctenidae, and Lycosidae (van Niekerk and Dippenaar‐Schoeman 1994; Lo et al. 2024; this study). Therefore, we removed Thomisidae and Lycosidae when reconstructing the ancestral state of oxyopid EG behaviors in the present study. To reconstruct the ancestral state and the evolutionary time of EG1–EG3 behaviors by lynx spiders, two different approaches were chosen and tested: BI and ML. We used RASP (Yu et al. 2014) for BI because it allowed for multistate Bayesian analysis of discrete states with the function Bayesian Binary MCMC (BBM) (Pagel 1999). The maximum number of states was limited to two. All other parameters were set to default settings. After discarding the first trees from trimmed trees obtained from BEAST analysis, we used the remaining 1000 trees for ancestral state reconstruction. The maximum clade credibility (MCC) tree produced in BEAST analysis was used as the input tree. On the basis of the dated chronogram for oxyopids, we pruned the tree to remove outgroups. ML analysis was performed in the R package Phytools (Revell 2012). We fit models of egg‐guarding behavior types under the equal rates (ER), symmetrical (SYM), and all rates different (ARD) models implemented using the ace function in ape. Results showed that the ER model was the best model for the maternal egg‐guarding pattern dataset. The ML tree of this study was used to construct the ancestral state, and each terminal taxon was marked with one of the following three characters: EG1, EG2, and EG3.

## 3 Results

### 3.1 Characteristics of Mitogenomes

Among the analyzed nine species and three genera of Oxyopidae, the largest mitogenome (14,890 bp in length) is from *Hamataliwa* ( *H. aurita* ), while the smallest (14,405 bp) is from *Oxyopes* ( *O. fujianicus* ) (Table 1). Within the genus *Oxyopes*, the sequence length of the largest mitogenome is only 14,477 bp ( *O. striagatus* ) (Table 1). *P. latikae* has a moderate mitogenome size (14,566 bp) (Table 1). The complete mitogenomes of the analyzed lynx spiders comprise 37 structural genes (13 PCGs, 22 tRNAs, and two rRNAs) and one control region (CR; Figure S1). These mitogenomes are biased toward A+T (75.2%–79.1%) and exhibit a similar nucleotide composition, negative AT‐skews, and positive GC‐skews (Table S4).

### 3.2 Phylogenetic Relationships

**FIGURE 1:**
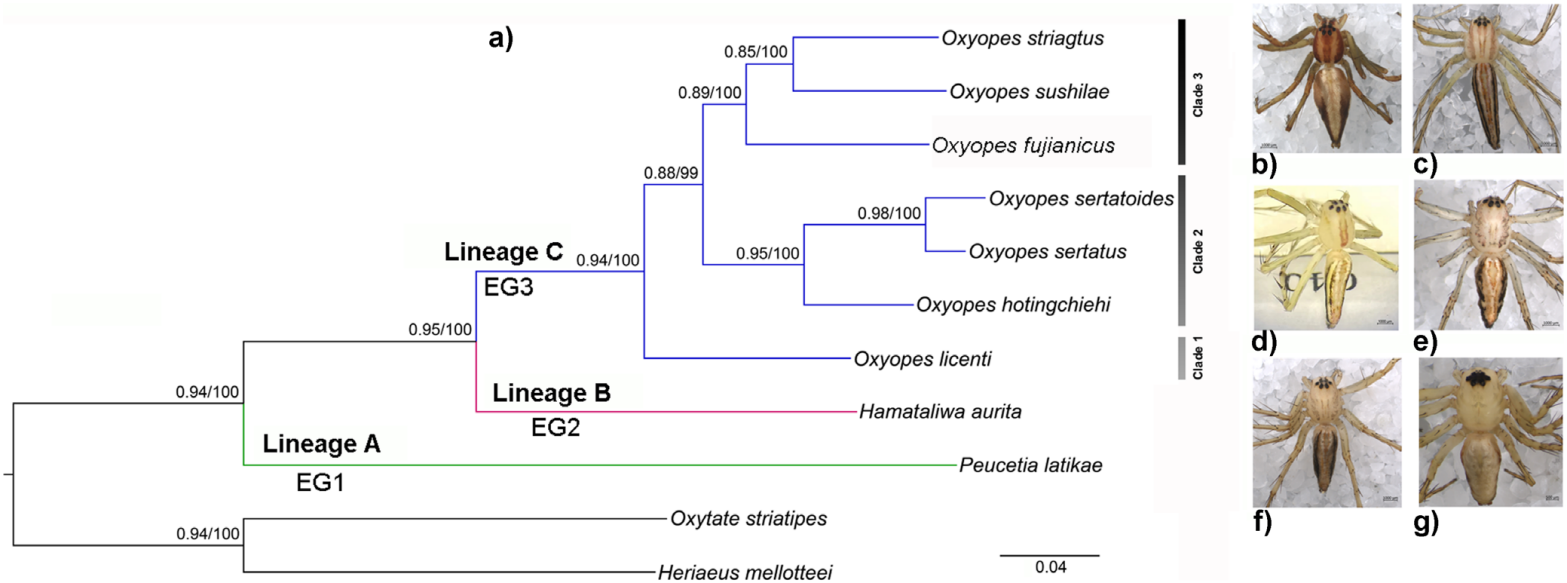
(a) Phylogenetic tree of the lynx spiders reconstructed using the concatenated 13 PCGs and two rRNAs dataset; (b) *O. striagtus*; (c) *O. sushilae* ; (d) *O. fujianicus* ; (e) *O. sertatoides* ; (f) *O. hotingchiehi* ; and (g) *H. aurita* . The numbers at the nodes represent support values from the Bayesian and maximum likelihood analyses.

**FIGURE 2:**
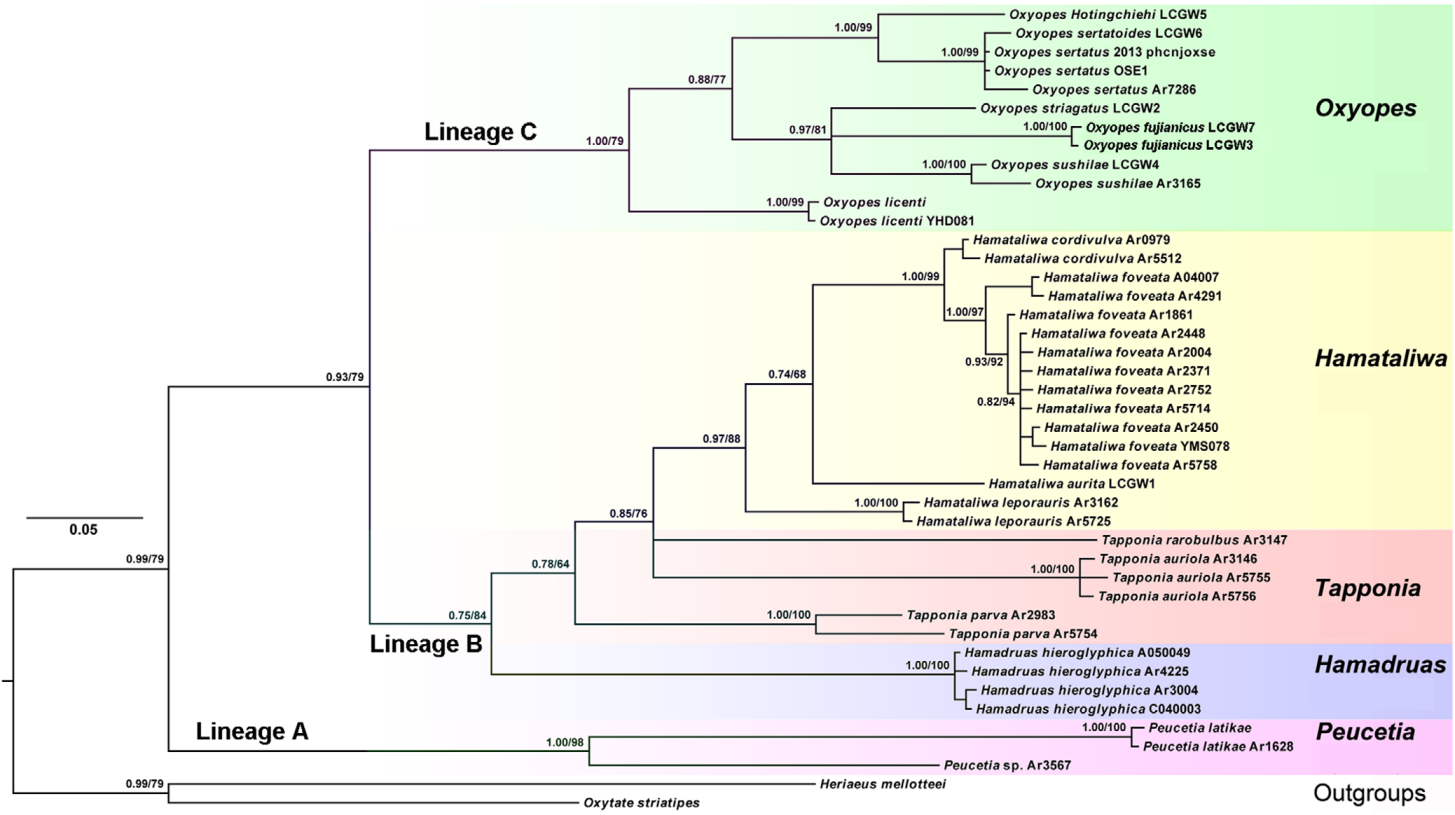
Phylogenetic tree of the lynx spiders reconstructed using the COX1 dataset. The numbers at the nodes represent support values from the Bayesian and maximum likelihood analyses.

Based on the concatenated 13 PCGs and two rRNAs sequences, the BI and ML analyses recovered the same topology (Figure 1; Figure S2). The genus *Hamataliwa* (Lineage B) is more closely related to *Oxyopes* (Lineage C) than to *Peucetia* (Lineage A). The genus *Oxyopes* forms a monophyletic group (posterior probability = 0.94, bootstrap support = 100%) containing three clades: Clade 1 only includes *O. licenti* ; Clade 2 (the *sertatus* group) consists of *O. sertatus* , *O. sertatoides* , and *O. hotingchiehi* ; and Clade 3 (the *striagatus* group) is composed of *O. striagatus* , *O. sushilae* , and *O. fujianicus* . The BI and ML phylogenies inferred from the COX1 dataset are also highly congruent (Figure 2; Figure S3). All sampled genera emerged as monophyletic and well‐supported groups, except *Tapponia*. The phylogenetic trees indicate that they also consist of three lineages: (1) Lineage A includes only *Peucetia* with the EG1 pattern; (2) Lineage B is composed of *Hamadruas*, *Tapponia*, and *Hamataliwa* with the EG2 pattern; and (3) Lineage C includes *Oxyopes* with the EG3 pattern.

### 3.3 Divergence Time

**FIGURE 3:**
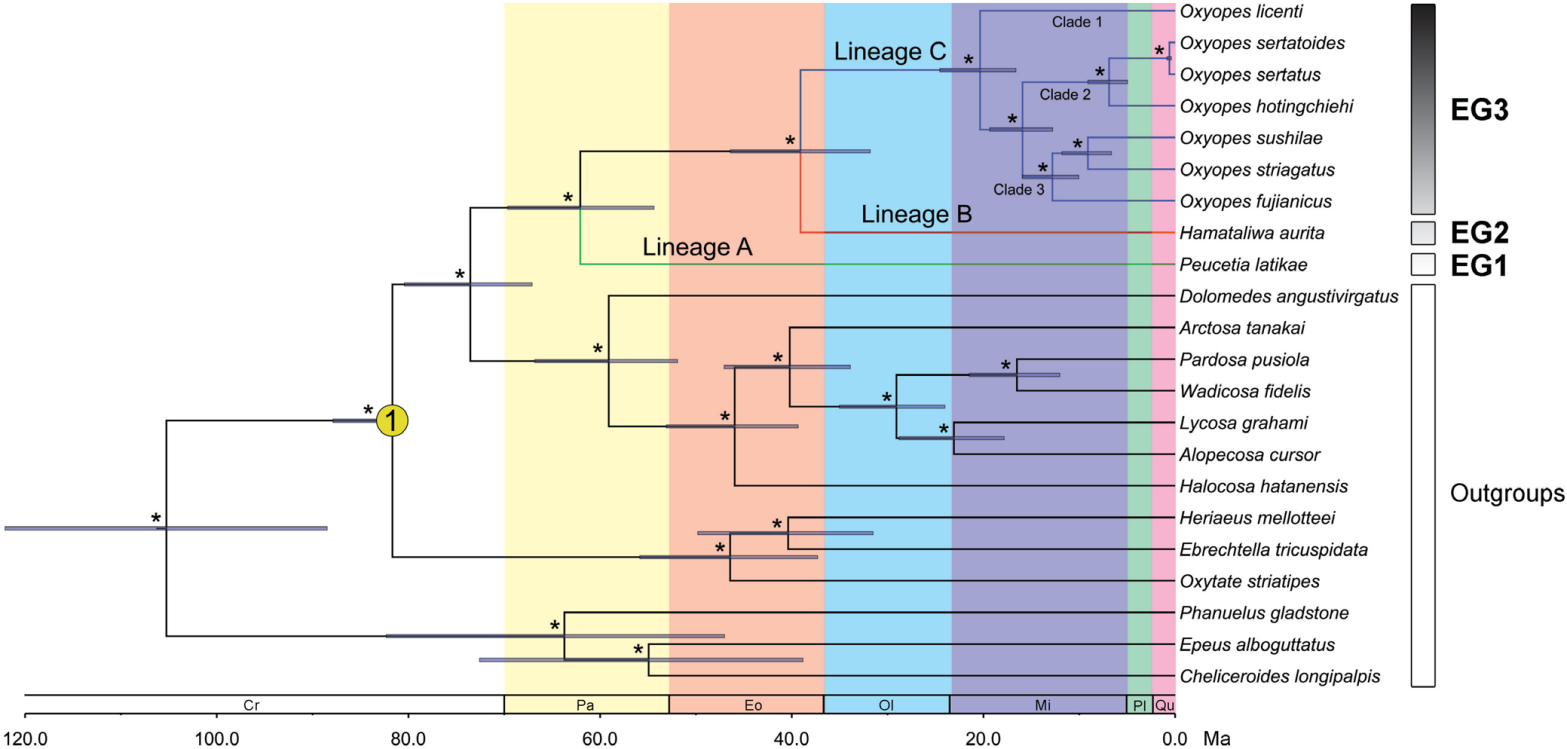
A dated phylogeny of the lynx spiders estimated using the concatenated 13 PCGs and two rRNAs dataset in BEAST. Yellow dot marks the calibration node (1 represents the divergent time of Oxyopidae and Thomisidae). “*” Indicates stable branches with Bayesian support > 0.95. Cr, Cretaceous; Eo, Eocene; Ma, million years ago; Mi, Miocene; Ol, Oligocene; Pa, Paleocene; Pl, Pliocene; Qu, Quaternary.

The origin of Oxyopidae was predicted to have occurred during the Late Cretaceous, around 73.5 (95% highest posterior density [95% HPD]: 67.1–80.4) Ma (Figure 3). Lineage A (*Peucetia*) diverged from Lineage B + Lineage C during the Paleocene, around 62.1 (95% HPD: 54.4–69.6) Ma (Figure 3). Lineage C (*Oxyopes*) was derived from a common ancestor during the Early Eocene/Late Oligocene, about 39.1 (95% HPD: 31.8–46.4) Ma, from which the genus initially diversified during the Late Oligocene, around 20.4 (95% HPD: 16.6–24.6) Ma (Figure 3).

### 3.4 Mitogenomic Evolution

**FIGURE 4:**
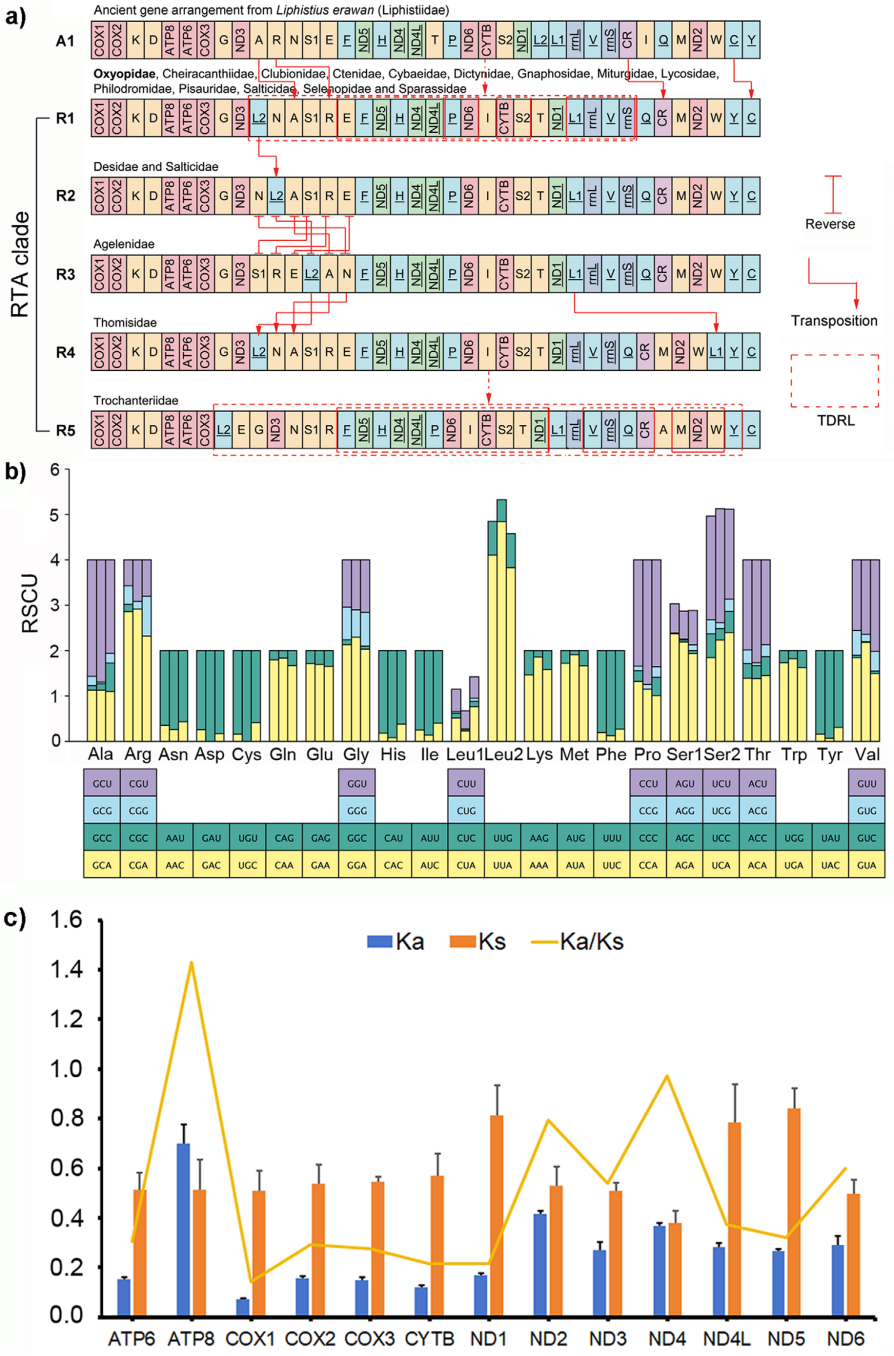
(a) The mechanism of gene rearrangement patterns in mitogenomes of the retrolateral tibial apophysis (RTA) spiders. Underlined genes are encoded in minor strand (N‐strand). PCGs and rRNAs are shown with standard abbreviations. PCGs and rRNAs are shown with standard abbreviations. The tRNA genes are abbreviated by one‐letter amino acid codes, L1 = CUN, L2 = UUR, S1 = AGN, and S2 = UCN. TDRL represents tandem duplication‐random loss. (b) Relative synonymous codon usage (RSCU) analyses of the lynx spiders. (c) The Ka and Ks values and Ka/Ks ratios of the concatenated 13 PCGs sequences for Oxyopidae.

Comparative analyses found the five gene rearrangement patterns (R1–R5) in the complete mitogenomes from 18 families within the RTA‐clade (Figure 4a). The analyzed nine species of oxyopids exhibit an identical gene arrangement (R1), which is also the most common rearrangement pattern in the RTA‐clade spiders (Figure 4a). The relative synonymous codon usage (RSCU) analyses indicate that the codon usage modes and major customarily utilized codons are highly conservative in the oxyopid mitogenomes (Figure 4b).

Among 13 PCGs of the lynx spiders, the average Ka value of ATP8 is the largest (Figure 4c), indicating that its evolutionary rate is the fastest. Both ND2 and ND4 also show a faster evolutionary rate, while the COX1 gene presents the slowest evolutionary rate (Figure 4c). The Ka/Ks value of only COX1 within the 13 PCGs is more than one (Figure 4c), indicating that the effect of natural selection on the change of the protein‐coding gene is positive.

### 3.5 Evolution of Maternal EG Behaviors

**FIGURE 5:**
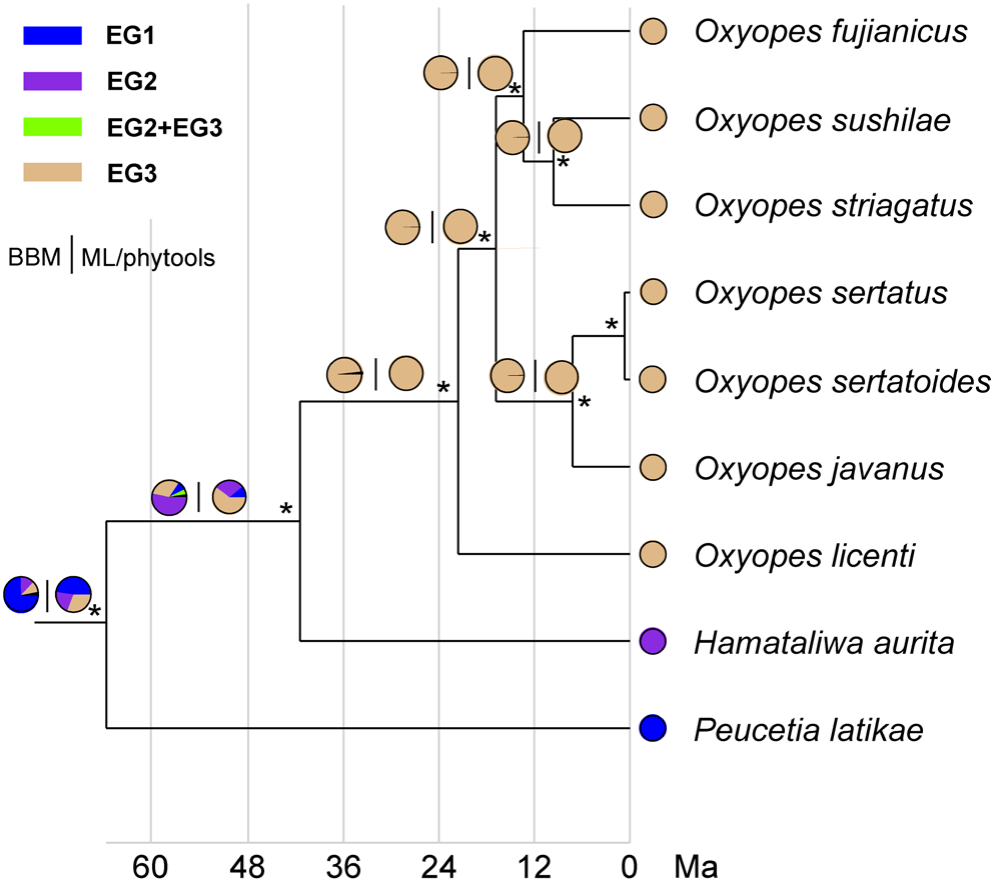
The ancestral state reconstructions of the maternal EG behaviors of lynx spiders based on the concatenated 13 PCGs and two rRNAs dataset. “*” Indicates stable branches with Bayesian support > 0.95. Ma, million years ago.

The ancestral state reconstructions of the oxyopid maternal EG behaviors suggest that the EG1 behavior firstly occurred during the Paleocene (Figure 5). The EG2 behavior subsequently emerged during the Eocene, and further the EG3 behavior rapidly yielded (Figure 5). Our results propose that the oxyopid maternal EG behaviors originated from EG1 and developed into EG2 and EG3.

## 4 Discussion

### 4.1 Phylogeny of Oxyopidae

The monophyly of Oxyopidae was supported by many studies of spider phylogeny (e.g., Polotow et al. 2015; Piacentini and Ramírez 2019; Kulkarni et al. 2023). Analyses found that Thomisidae, Ctenidae, or Senoculidae might be the sister group of Oxyopidae (Polotow et al. 2015; Wheeler et al. 2017; Fernández et al. 2018; Kulkarni et al. 2023). The phylogenetic relationships among taxa within the family are less studied. Townsend and Felgenhauer (2001) examined the distribution and shape of cuticular scales from oxyopid species and indicated that the family had three distinct lineages: (1) *Peucetia*, *Tapinillus*, and *Schaenicoscelis*; (2) *Hamataliwa* and *Tapponia*; (3) *Oxyopes* and *Hostus*. Our molecular phylogenetic analyses also suggest that Oxyopidae includes three lineages: Lineage A (*Peucetia*), Lineage B (*Hamadruas*, *Tapponia*, and *Hamataliwa*), and Lineage C (*Oxyopes*), and Lineage B is closer to Lineage C than to Lineage A. Lo et al. (2024) proposed that *Hamadruas* was closer to *Oxyopes* than to *Tapponia + Hamataliwa*. However, in this study, *Hamadruas*, *Tapponia*, and *Hamataliwa* are closely related. That is also supported by the evidence of the male palp morphology (with tegular lobe and cymbium basal apophysis), habitat preference (canopy or understory layer), and egg sac guarding behavior (egg sac is attached to a leaf hung on silk). *Tapponia* is a polyphyletic group, and the genus is sister to the monophyletic *Hamataliwa*, which is consistent with the molecular results of Lo et al. (2024). Morphologically, Deeleman‐Reinhold (2004) also pointed out that the copulatory organs of the two genera appeared to be conservative and uniform, whereas their somatic characters were much more diverse. Based on the mitogenomic data, the inferred phylogenetic relationships among species of *Oxyopes* are supported by their morphology (Song et al. 1999; Lo et al. 2024). For example, *O. licenti* genetically diverged from the sister *sertatus* and *striagatus* groups. Morphologically, this species differs from both species groups by the peach‐like sclerite in the middle of the epigyne (Song et al. 1999). Our results provide a relatively holistic framework and valuable data toward the future resolution of phylogenetic relationships in Oxyopidae.

### 4.2 Evolution of the Oxyopid Mitogenomes

Mitochondrial gene rearrangement is one of the most important evolutionary characteristics for certain arthropod taxa, including Araneae (Boore et al. 1998; Cameron 2014; Zhang et al. 2021; Li et al. 2022). From our analyses, only one common rearrangement pattern (R1) in the RTA spiders was found for the nine species and three genera of Oxyopidae, and the gene rearrangement highly deviates from the presumed ancestral pattern ( *Liphistius erawan* Schwendinger, 1996). We found that the oxyopid PCGs displayed the different Ka/Ks values, and the value of only the COX1 was more than one, indicating the gene change was positively affected by natural selection. These results were also observed in other animals such as hynobiids (Zhang et al. 2022).

### 4.3 Maternal EG Behavioral Evolution of Lynx Spiders

The maternal egg‐guarding (EG) behaviors of spiders have evolved as a strategy of life history to improve their reproductive success, particularly in cursorial groups such as Oxyopidae and its sisters Thomisidae, Ctenidae and Lycosidae facing high costs (Murphy et al. 2006; Foelix 2011; Yip and Rayor 2014). The strategies of maternal EG behaviors have different levels and diverse forms (EG1–EG3) in the oxyopid genera *Peucetia*, *Hamadruas*, *Hamataliwa*, *Tapponia*, and *Oxyopes*, and these EG behaviors differ from those of the sister groups Thomisidae and Ctenidae (van Niekerk and Dippenaar‐Schoeman 1994; Lo et al. 2024; this study). Other oxyopid genera *Hostus*, *Pseudohostus*, *Schaenicoscelis*, and *Tapinillus* have not yet been reported for their EG behaviors. Due to the unavailable samples of other oxyopid taxa, this study only analyzed the maternal EG behavioral evolution of *Peucetia* (one species), *Hamataliwa* (one species), and *Oxyopes* (seven species) using ancestral state reconstruction methods and proposed that the EG1 behavior of *Peucetia* (van Niekerk and Dippenaar‐Schoeman 1994) seemed to be primitive and probably occurred during the Paleocene. The EG2 behaviors of *Hamadruas*, *Hamataliwa*, and *Tapponia* and the EG3 behavior of the recently rapidly diversified *Oxyopes* were derived from the EG1 behavior. We speculated that the EG behavior evolution of lynx spiders was likely the consequence of the trade‐off between egg safety and female energy consumption.

### 4.4 Future Directions

This study has advanced our understanding of the phylogenetic relationships among partial species/groups of Oxyopidae and the evolutionary pattern of their maternal EG behaviors using the phylogenetic methods based on mitogenomics. However, to obtain a robust phylogeny of this family, it is necessary to expand the sampling range to include more species in the globe in order to reveal deeper levels of phylogenetic relationships and EG behavioral evolution of Oxyopidae. Additionally, integrating different types of data, such as transcriptome and UCEs, is crucial. By utilizing morphological, biological, ecological, and molecular data and conducting multidimensional phylogenetic analyses, we can improve the accuracy of phylogenetic research. Such integrative approaches will provide a more robust framework for understanding the phylogeny and evolution within Oxyopidae.

## Author Contributions

**Dan Fu:** conceptualization (equal), data curation (equal), formal analysis (equal), investigation (equal), methodology (equal), resources (equal), software (equal), validation (equal), writing – original draft (equal), writing – review and editing (equal). **Lijuan Liu:** conceptualization (equal), data curation (equal), formal analysis (equal), resources (equal), software (equal), writing – original draft (equal). **Changjun Wu:** data curation (equal), resources (equal). **Yufa Luo:** conceptualization (lead), funding acquisition (equal), investigation (equal), project administration (equal), supervision (equal), validation (equal), writing – review and editing (equal).

## Ethics Statement

The authors have nothing to report.

## Conflicts of Interest

The authors declare no conflicts of interest.

## Supporting information


**Data S1:** ece372192‐sup‐0001‐DataS1.zip.

## Data Availability

All of the data that support the findings of this study is available in the Supporting Information.
