# Supplementary material for: Mitogenomic Insights on the Phylogeny and Evolution of Lynx Spiders (Araneae, Oxyopidae)
Source: Ecol Evol. 2025 Sep 18;15(9):e72192. doi: 10.1002/ece3.72192 (PMC12445203; doi:10.1002/ece3.72192)
Supplement: Supplementary file 1 — Data S1: ece372192‐sup‐0001‐DataS1.zip. [file ECE3-15-e72192-s001.zip › The renewed version_ece372192-sup-0001-DataS1/The renewed version_Supporting information.docx]

**Supporting Information**

**Mitogenomic insights on the phylogeny and evolution of lynx spiders (Araneae: Oxyopidae)**

Dan Fu, Lijuan Liu, Changjun Wu and Yufa Luo

Key Laboratory of Wetland Biodiversity of the Jianhu Basin of Shaoxing, School of Life and Environmental Sciences, Shaoxing University, Shaoxing 312000, China

Corresponding author: Yufa Luo ([lyf223@](mailto:lyf223@)126.com)

**Table of Contents:**

| **Table S1** | **Pages 2-5** |
| --- | --- |
| **Table S2** | **Page 6** |
| **Table S3** | **Page 7** |
| **Figure S1** | **Page 8** |
| **Figure S2** | **Page 9** |
| **Figure S3** | **Page 10** |

**Table S1**. Samples used in this study: taxon name, specimen voucher, sample collection locality, and GenBank accession numbers.

| Taxon | Family | Voucher | Locality | Mitogenome/COX1 | References |
| --- | --- | --- | --- | --- | --- |
| *Oxyopes sushilae* | Oxyopidae | Ar3165 | China, Taiwan | PP054131 | Lo et al. 2024 |
| *Oxyopes sushilae* | Oxyopidae | LCGW4 | China, Jiangxi (N:25°46′21″, E: 114°58′10″) | PV692094 | This study |
| *Oxyopes hotingchiehi* | Oxyopidae | LCGW5 | China, Jiangxi (N:25°46′21″, E: 114°58′10″) | PV692091 | This study |
| *Oxyopes striagatus* | Oxyopidae | LCGW2 | China, Jiangxi (N:25°45′27″, E: 114°58′12″) | PV692093 | This study |
| *Oxyopes fujianicus* | Oxyopidae | LCGW3 | China, Jiangxi (N:25.45′33″, E: 114°57′24″) | PV692090 | This study |
| *Oxyopes fujianicus* | Oxyopidae | LCGW7 | China, Jiangxi (N:25°45′27″, E: 114°58′11″) | *submitting* | This study |
| *Oxyopes sertatoides* | Oxyopidae | LCGW6 | China, Jiangxi (N:25°45′33″, E: 114°57′24″) | PV692092 | This study |
| *Oxyopes sertatus* | Oxyopidae | 2013 phcnjoxse | China, Nanjing | KM272950 | Pan et al. 2016 |
| *Oxyopes sertatus* | Oxyopidae | OSE1 | China | KY467132 | Wang et al. 2018 |
| *Oxyopes sertatu* | Oxyopidae | Ar7286 | China, Taiwan | PP054130 | Lo et al. 2024 |
| *Oxyopes licenti* | Oxyopidae | - | China, Gansu | MT741489 | Li et al. 2022 |
| *Oxyopes licenti* | Oxyopidae | YHD081 | China | OQ884071 | Unpublished |
| *Hamataliwa cordivulva* | Oxyopidae | Ar0979 | China, Taiwan | PP054080 | Lo et al. 2024 |
| *Hamataliwa cordivulva* | Oxyopidae | Ar5512 | China, Taiwan | PP054084 | Lo et al. 2024 |
| *Hamataliwa leporauris* | Oxyopidae | Ar3162 | China, Taiwan | PP054115 | Lo et al. 2024 |
| *Hamataliwa leporauris* | Oxyopidae | Ar5725 | China, Taiwan | PP054116 | Lo et al. 2024 |
| *Hamataliwa aurita* | Oxyopidae | LCGW1 | China, Jiangxi (N:27°45′12″, E: 117°40′36″) | PV743049 | This study |
| *Hamataliwa foveata* | Oxyopidae | A04007 | China, Taiwan | PP054086 | Lo et al. 2024 |
| *Hamataliwa foveata* | Oxyopidae | Ar4291 | China, Taiwan | PP054100 | Lo et al. 2024 |
| *Hamataliwa foveata* | Oxyopidae | Ar1861 | China, Taiwan | PP054087 | Lo et al. 2024 |
| *Hamataliwa foveata* | Oxyopidae | Ar2448 | China, Taiwan | PP054090 | Lo et al. 2024 |
| *Hamataliwa foveata* | Oxyopidae | Ar2004 | China, Taiwan | PP054088 | Lo et al. 2024 |
| *Hamataliwa foveata* | Oxyopidae | Ar2371 | China, Taiwan | PP054089 | Lo et al. 2024 |
| *Hamataliwa foveata* | Oxyopidae | Ar2752 | China, Taiwan | PP054093 | Lo et al. 2024 |
| *Hamataliwa foveata* | Oxyopidae | Ar5714 | China, Taiwan | PP054104 | Lo et al. 2024 |
| *Hamataliwa foveata* | Oxyopidae | Ar2450 | China, Taiwan | PP054091 | Lo et al. 2024 |
| *Hamataliwa foveata* | Oxyopidae | YMS078 | China, Taiwan | PP054112 | Lo et al. 2024 |
| *Hamataliwa foveata* | Oxyopidae | Ar5758 | China, Taiwan | PP054106 | Lo et al. 2024 |
| *Tapponia parva* | Oxyopidae | Ar2983 | China, Taiwan | PP054162 | Lo et al. 2024 |
| *Tapponia parva* | Oxyopidae | Ar5754 | China, Taiwan | PP054163 | Lo et al. 2024 |
| *Tapponia auriola* | Oxyopidae | Ar3146 | China, Taiwan | PP054159 | Lo et al. 2024 |
| *Tapponia auriola* | Oxyopidae | Ar5755 | China, Taiwan | PP054160 | Lo et al. 2024 |
| *Tapponia auriola* | Oxyopidae | Ar5756 | China, Taiwan | PP054161 | Lo et al. 2024 |
| *Tapponia rarobulbus* | Oxyopidae | Ar3147 | China, Taiwan | PP054164 | Lo et al. 2024 |
| *Hamadruas hieroglyphica* | Oxyopidae | A05-0049 | China, Taiwan | PP054057 | Lo et al. 2024 |
| *Hamadruas hieroglyphica* | Oxyopidae | Ar4225 | China, Hong Kong | PP054067 | Lo et al. 2024 |
| *Hamadruas hieroglyphica* | Oxyopidae | Ar3004 | China, Taiwan | PP054066 | Lo et al. 2024 |
| *Hamadruas hieroglyphica* | Oxyopidae | C040003 | China, Taiwan | PP054074 | Lo et al. 2024 |
| *Peucetia latikae* | Oxyopidae | - | China, Hunan | MW832846 | Li et al. 2022 |
| *Peucetia latikae* | Oxyopidae | Ar1628 | China, Taiwan | PP054133 | Lo et al. 2024 |
| *Peucetia* sp. | Oxyopidae | Ar3567 | China, Taiwan | PP054155 | Lo et al. 2024 |
| *Oxytate striatipes* | Thomisidae | - | - | KM507783 | Kim et al. 2016 |
| *Heriaeus mellotteei* | Thomisidae | - | China, Gansu | MW832850 | Li et al. 2022 |
| *Ebrechtella tricuspidata* | Thomisidae | - | China | KU852748 | Zhu et al. 2019 |
| *Dolomedes angustivirgatus* | Pisauridae | - | China, Zhejiang (N:30°03'00", E:121°09'00") | KU354434 | Wang et al. 2020 |
| *Cheliceroides longipalpis* | Salticidae | - | - | MH891570 | Chen et al. 2018 |
| *Epeus alboguttatus* | Salticidae | - | - | MH922026 | Yang et al. 2018 |
| *Phanuelus gladstone* | Salticidae | MCC-ARA-SR668-4A | India, Tamil Nadu | MT773150 | Unpublished |
| *Alopecosa cursor* | Lycosidae | - | China, Gansu | MW776435 | Li et al. 2022 |
| *Arctosa tanakai* | Lycosidae | - | China, Chongqing (N:29°49'47", E:106°21'26") | CNA0013709 | Zhao et al. 2018 |
| *Halocosa hatanensis* | Lycosidae | - | China: Xinjiang | MT174468 | Chen et al. 2020 |
| *Lycosa grahami* | Lycosidae | - | China, Yunnan (N:24°04'00", E:101°57'00") | ON951647 | Ye et al. 2023 |
| *Pardosa pusiola* | Lycosidae | - | China, Guangdong (N:24°14'30", E:112°53'56") | ON118383 | Yi et al. 2023 |
| *Wadicosa fidelis* | Lycosidae | - | China, Zhejiang (N:30°03'00", E:121°09'00") | KP100666 | Wang et al. 2016 |

**References**

Chen C, Xu K, Yan Y, Yang W, Yang H, Li C, Yang D (2018) The complete mitochondrial genome of a jumping spider, *Cheliceroides longipalpis* Zabka (Araneae: Salticidae). Mitochondrial DNA Part B 4(1): 95–96.

Chen JY, Wu RB, Zhang ZS (2020) The complete mitochondrial genome of *Halocosa hatanensis* (Araneae: Lycosidae). Mitochondrial DNA Part B 5(3): 3178–3179.

Kim JY, Yoo JS, Park YC (2016) The complete mitochondrial genome of the green crab spider *Oxytate striatipes* (Araneae: Thomisidae). Mitochondrial DNA Part A 27(3): 1878–1879.

Li M, Chen WT, Zhang QL, Liu M, Xing CW, Cao Y, Luo FZ, Yuan ML (2022) Mitochondrial phylogenomics provides insights into the phylogeny and evolution of spiders (Arthropoda: Araneae). Zoological Research 43(4): 566–584.

Lo YY, Cheng RC, Lin CP (2024) Integrative species delimitation and five new species of lynx spiders (Araneae, Oxyopidae) in Taiwan. PLoS One 19(5): e0301776.

Pan WJ, Fang HY, Zhang P, Pan HC (2016) The complete mitochondrial genome of striped lynx spider *Oxyopes sertatus* (Araneae: Oxyopidae). Mitochondrial DNA Part A 27(3): 1616–1617.

Wang ZL, Huang J, Li MY, Yu XP (2020) The complete mitochondrial genome of a nursery-web spider *Dolomedes angustivirgatus*(Araneae: Pisauridae). Mitochondrial DNA Part B 5(2): 1695–1696.

Wang ZL, Li C, Fang WY, Yu XP (2016) The complete mitochondrial genome of the wolf spider *Wadicosa fidelis* (Araneae: Lycosidae). Mitochondrial DNA Part A 27(6): 3909–3910.

Wang ZL, Yang XQ, Wang TZ, Yu X (2018) Assessing the effectiveness of mitochondrial COI and 16S rRNA genes for DNA barcoding of farmland spiders in China. Mitochondrial DNA Part A 29(5): 695–702.

Yang D, Yan X, Xu K, Yang W, Li C (2018) The complete mitochondrial genome of *Epeus alboguttatus* (Araneae: Salticidae). Mitochondrial DNA Part B 4(1): 316–317.

Ye W, Zhao X, Xu T, Liu H (2023) Complete mitochondrial genomes of *Lycosa grahami* and *Lycosa sp.* (Araneae: Lycosidae): comparison within the family Lycosidae. International Journal of Tropical Insect 43: 533–545.

Yi J, Liu M, Liu J, Mao Y, Lin M, Xu H, An Y, Wu H, Li J (2023) The complete mitochondrial genome of *Pardosa pusiola* (Araneae, Lycosidae) and its phylogenetic implications. Entomological Research 53: 148–157.

Zhao HY, Zhang ZS (2018) The complete mitochondrial genome of *Arctosa tanakai* (Araneae: Lycosidae). Acta Arachnologica Sinica 27(1): 57–64.

Zhu HF, Wang ZY, Wang ZL, Yu XP (2019) Complete mitochodrial genome of the crab spider *Ebrechtella tricuspidata* (Araneae: Thomisidae): A novel tRNA rearrangement and phylogenetic implications for Araneae. Genomics 111(6): 1266–1273.

**Table S2**. Parameters of the best-fitting substitution model for each gene partition selected under the Bayesian information criterion (BIC) in the Bayesian inference.

| Gene | Model | Partition | -InL | p | BIC | deltaBIC | Weight | Cumweight |
| --- | --- | --- | --- | --- | --- | --- | --- | --- |
| 12S | GTR+G | 012345 | 3011.6361 | 29 | 6211.3266 | 0.0 | 0.7842 | 0.784 |
| 16S | GTR+G | 012345 | 4355.0283 | 29 | 8909.4384 | 0.0 | 0.894 | 0.894 |
| ATP6 | HKY+I+G | 010010 | 2735.9926 | 26 | 5640.2662 | 0.0 | 0.5014 | 0.501 |
| ATP8 | HKY+G | 010010 | 1009.9774 | 25 | 2144.7156 | 0.0 | 0.9048 | 0.905 |
| COI1 | GTR+I+G | 012345 | 5784.4067 | 30 | 11788.8237 | 0.0 | 0.9801 | 0.98 |
| COI2 | GTR+G | 012345 | 2934.0608 | 29 | 6056.6154 | 0.0 | 0.9289 | 0.929 |
| COI3 | GTR+G | 012345 | 3388.0627 | 29 | 6968.6817 | 0.0 | 0.7585 | 0.758 |
| Cytb | GTR+I+G | 012345 | 4362.6744 | 30 | 8934.3579 | 0.0 | 0.6135 | 0.614 |
| ND1 | GTR+I+G | 012345 | 4237.4255 | 30 | 8678.1462 | 0.0 | 0.9864 | 0.986 |
| ND2 | GTR+G | 012345 | 5087.703 | 29 | 10372.5787 | 0.0 | 0.925 | 0.925 |
| ND3 | HKY+G | 010010 | 1667.1572 | 25 | 3477.6482 | 0.0 | 0.8957 | 0.896 |
| ND4 | GTR+I+G | 012345 | 6551.2224 | 30 | 13315.7412 | 0.0 | 0.8068 | 0.807 |
| ND4L | HKY +G | 010010 | 1237.4465 | 25 | 2612.5263 | 0.0 | 0.7342 | 0.734 |
| ND5 | GTR+I+G | 012345 | 8877.5921 | 30 | 17976.1016 | 0.0 | 0.9813 | 0.981 |
| ND6 | GTR+G | 012345 | 2277.3682 | 29 | 4729.6959 | 0.0 | 0.4697 | 0.47 |

**Table S3**. The details and assignments of the calibration point used in BEAST analysis.

| Taxa | BEAST | | | | | MCMC | |
| --- | --- | --- | --- | --- | --- | --- | --- |
|  |  | Distribution | Mean | Stdev | Offset | Minimum | Maximum |
| Oxyopidae + Thomisidae | crow | logNormal | 0.6 | 1.0 | 80 | 80 | 90 |

Notes: The time units of all parameters were set in million years.

**Table S4.** Complete mitogenome content and skewness of the nine oxyopid species.

| Species | A (%) | T (%) | G (%) | C (%) | A+T (%) | AT-skew | GC-skew |
| --- | --- | --- | --- | --- | --- | --- | --- |
| *Peucetia latikae* | 32.49 | 42.75 | 16.25 | 8.51 | 75.24 | -0.14 | 0.31 |
| *Hamataliwa aurita* | 35.27 | 43.85 | 12.90 | 7.99 | 79.11 | -0.11 | 0.24 |
| *Oxyopes licenti* | 35.00 | 43.08 | 13.67 | 8.25 | 78.07 | -0.10 | 0.25 |
| *Oxyopes hotingchiehi* | 33.71 | 42.69 | 15.21 | 8.39 | 76.39 | -0.12 | 0.29 |
| *Oxyopes sertatus* | 33.05 | 42.89 | 15.90 | 8.16 | 75.94 | -0.13 | 0.32 |
| *Oxyopes sertatoides* | 33.12 | 43.03 | 15.74 | 8.11 | 76.15 | -0.13 | 0.32 |
| *Oxyopes fujianicus* | 35.13 | 42.46 | 14.04 | 8.37 | 77.58 | -0.09 | 0.25 |
| *Oxyopes sushilae* | 35.77 | 43.08 | 12.96 | 8.19 | 78.84 | -0.09 | 0.23 |
| *Oxyopes striagatus* | 34.59 | 42.56 | 14.45 | 8.40 | 77.15 | -0.10 | 0.26 |


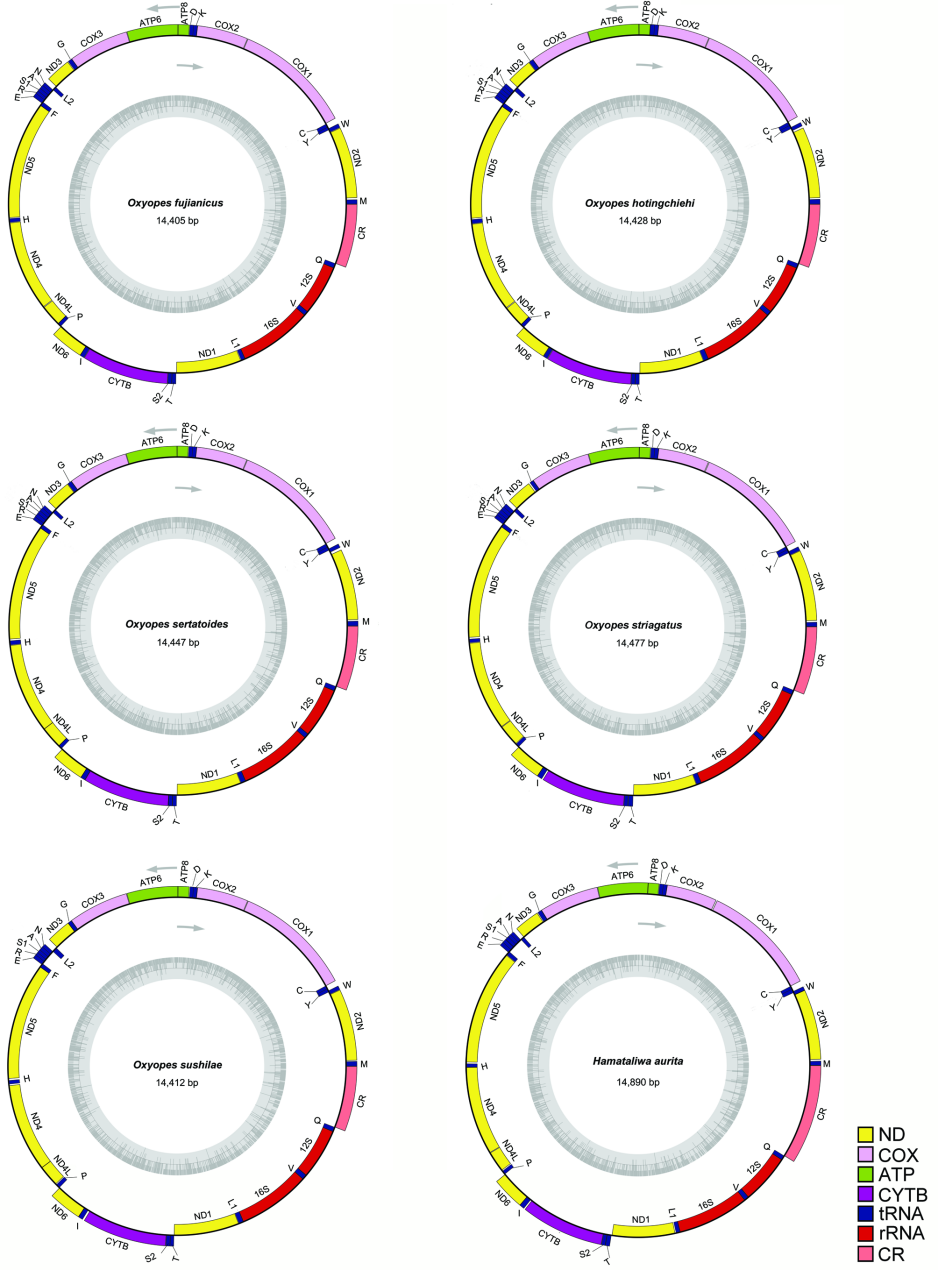


**Figure S1.** Characteristics of the oxyopid mitogenomes sequenced in this study.


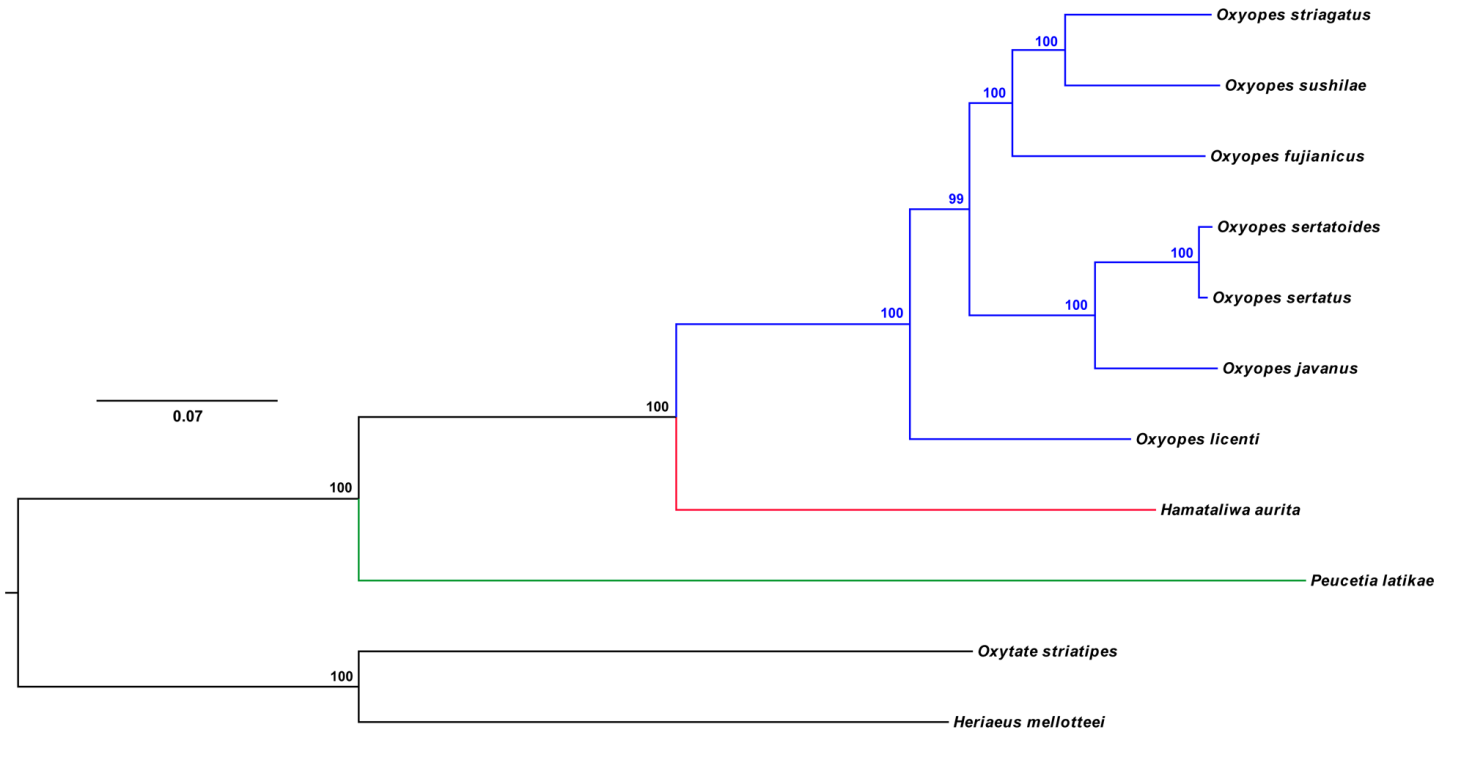


**Figure S2.** The ML tree of lynx spiders reconstructed using the combined 13 PCG and two rRNA sequences. The numbers at the nodes represent support values from the ML analysis.


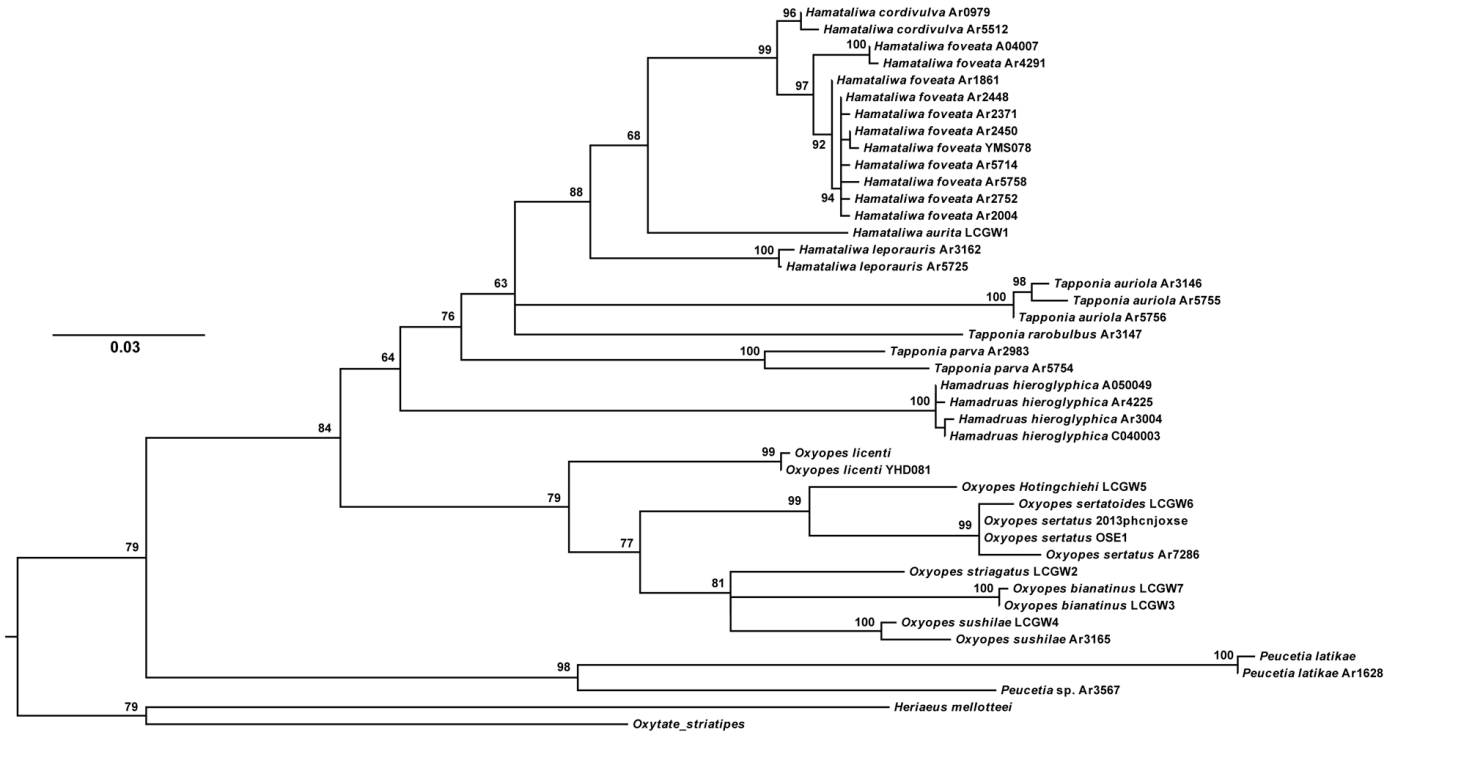


**Figure S3.** The ML tree of lynx spiders reconstructed using the *COX1* sequences. The numbers at the nodes represent support values from the maximum likelihood analysis.
